# Action Planning for Reducing Sugar-Sweetened Beverage Intake in Appalachian Adults: Longitudinal Process Evaluation of a Digital Behavioral Health Intervention

**DOI:** 10.2196/71241

**Published:** 2026-06-12

**Authors:** Annie Reid, Lee Ritterband, Kathleen Porter, Donna-Jean Brock, Christina Frederick, Jamie Zoellner

**Affiliations:** 1 Department of Public Health Sciences, School of Medicine, University of Virginia, 16 E Main St, Christiansburg, VA, 24073, United States, 1 434-962-4488; 2 Department of Psychiatry and Neurobehavioral Sciences, School of Medicine, University of Virginia, Charlottesville, VA, United States

**Keywords:** digital health intervention, action planning, sugar-sweetened beverages, behavior change, process evaluation

## Abstract

**Background:**

Digital health interventions show promise for promoting behavior change, but how they incorporate action planning strategies is underreported. This oversight limits understanding of how to implement behavior change techniques. iSIPsmarter is a digital health intervention aimed at reducing sugar-sweetened beverage (SSB) consumption among Appalachian adults.

**Objective:**

This study aimed to examine the digital action planning process in the iSIPsmarter intervention, specifically by (1) assessing the frequency of action plan engagement, (2) evaluating participants’ perceived difficulty implementing their action plans and examining progress toward achieving SSB reduction and weight goals, and (3) exploring the selection of barriers and strategies.

**Methods:**

The digital action planning process is embedded within 5 of iSIPsmarter’s 6 behavioral content modules (Cores) and paired with self-monitoring of SSB intake via SMS text messaging and weight via a cellular-enabled scale. Participants first self-select program goals for SSB intake (in ounces) and weight (loss or maintenance). Then, in Cores 2‐6, they complete action plans using personalized tracking feedback, recommendations, and goal progress updates. Participants identify barriers and strategies using preprogrammed or write-in responses. Summary statistics described the aims.

**Results:**

Participants (n=119) were predominately White (Caucasian), female, aged between 18 and 44 years, college-educated, and from rural counties. On average, participants completed 4.5 (SD 1.1) of 5 possible SSB action plans, with 80% (95/119) completing all 5. Across all Cores, perceived difficulty implementing action plans and achieving goals remained relatively stable, with an average of 48% rating the tasks as impossible or hard, 29% as neither hard nor easy, and 24% as easy or very easy. Nearly half achieved their self-selected weekly SSB goals, and one-third made progress toward them. At Core 6, 57% (54/95) of participants met their self-selected SSB program goal, while 46% (44/95) met the recommended SSB intake of less than 8 ounces per day. Of 119 participants, 53 (45%) modified their SSB barriers, and 63 (53%) selected new strategies during action planning. Top reported SSB barriers included (1) caffeine, (2) taste, and (3) habit. Among those with a program weight loss goal (n=94), the average weight loss was −1.3% (SD 2.6) at the 9-week follow-up, with 61% (57/94) achieving their goal. By 6 months, weight loss increased to −2.1% (SD 5.6), with 54% (49/90) achieving their goal. Of those completing weight action plans, 62% (69/112) modified their barriers. Top weight barriers included (1) sweets, (2) portion sizes, and (3) eating healthy foods.

**Conclusions:**

Findings underscore the value of digital action planning as a central behavior change technique within a nutrition-focused digital intervention. High action plan completion and consistent strategy adaptation suggest that structured, digitally personalized goal setting and action planning processes can effectively support behavior change, particularly among underserved populations with limited access to preventative care.

## Introduction

### Background

Digital technologies, which include web programs, mobile apps, and SMS text messaging, are increasingly being used within behavior change interventions. Their widespread availability marks a promising shift in the field, offering improvements in accessibility and personalized support. This is especially important for hard-to-reach populations, such as those in rural or underserved areas, who often face barriers to traditional in-person interventions [1]. By leveraging digital platforms, these interventions can extend their reach and improve health outcomes in populations with limited access to preventive care [2].

Behavioral interventions often use behavior change techniques (BCTs), which provide structured strategies targeting specific behavioral mechanisms [3]. Michie and colleagues’ [4] well-recognized taxonomy of 93 distinct BCTs offers a comprehensive set of evidence-based strategies to facilitate behavior change. While BCTs are often used in behavior change interventions, there is limited understanding of their application and impact within digital health interventions. Similarly, although action planning is a key BCT associated with improved outcomes in behavioral interventions [5,6], its use and impact within digital health interventions remain underexplored and underutilized.

Action planning involves creating a detailed and concrete plan for how and when a specific behavior will be performed, including details on the context, frequency, duration, and intensity of the behavior [4]. Among BCTs, action planning is particularly vital because it helps individuals translate behavior change intentions into actionable steps, effectively bridging the gap between motivation and sustained behavior change. Likewise, action planning functions as the central BCT that integrates self-monitoring, goal setting, and problem-solving by providing a structured technique for translating participants’ goals into concrete plans through barrier identification and strategy selection [4,7]. Given its central role in facilitating the implementation of behavior change intentions, action planning has been consistently associated with improved outcomes across a range of health behaviors, making it a key focus for behavior change intervention design and evaluation [7,8].

A recent umbrella review of systematic reviews and meta-analyses identified the most effective BCTs at improving health outcomes among adults within digital health interventions targeting the prevention and management of noncommunicable disease. The review concluded that action planning, goal setting, feedback and monitoring, and personalization should be prioritized for inclusion in digital health interventions [9]. Yet, only 15% (13/85) of systematic reviews identified digital health intervention studies using action planning, and the included studies rarely provided sufficient detail on how action planning was implemented. This lack of use and limited reporting create a significant gap in understanding how to design and use action planning components effectively in digital health contexts. Addressing this gap in both implementation and reporting of digital action planning strategies is essential to improving the design of digital health interventions and ensuring that high-impact BCTs are used effectively to support behavior change and improve health outcomes.

### Digital Health Interventions and Sugar-Sweetened Beverage Context in Rural Appalachia (United States)

As the digital divide—the gap between those with and with no reliable access to internet-connected technology [10]—continues to narrow, digital health interventions have become increasingly vital for rural communities like those in the Appalachian region of the United States [11,12]. Appalachia faces numerous barriers to accessing behavioral health interventions, including transportation challenges, limited infrastructure for health care, and socioeconomic constraints [13]. Compounding these issues are the high prevalence of sugar-sweetened beverage (SSB; eg, soda, sweet tea, sports and energy drinks, and fruit drinks) consumption and SSB-related health outcomes, including obesity and weight-related conditions [13,14]. In southwest Virginia, a part of Appalachia and the focus region of this study, SSB intake is more than double the national average and more than 4 times the recommended daily amount of 8 ounces or less per day [15,16]. Importantly, in recent years, strides have been made in bridging the digital divide as access to broadband and greater device ownership throughout Appalachia has increased [17,18]. Thus, given the relatively low cost and high scalability of digital health interventions, they offer promise in overcoming barriers to accessing behavioral interventions and improving nutrition-related health outcomes in the underserved Appalachian region. Although a recent review identified several digital behavioral interventions that incorporated action planning, none specifically targeted nutrition-related behaviors [9]. Notably, one promising feasibility study has begun to address this gap, highlighting the need for further research in this area [19].

iSIPsmarter, the program of focus of this paper, is a theory-based, digital behavioral health intervention designed to reduce SSB intake among Appalachian adults [20]. Using a randomized controlled trial (RCT), iSIPsmarter was shown to be effective in reducing SSB ounces and in improving weight compared with a static participant education comparison group [21]. Also, intervention engagement was high, and no significant relationship was found between engagement levels and outcomes. However, knowing that action planning is a vital self-regulatory BCT, providing detail and examining its role explicitly will help not only in better understanding the success of iSIPsmarter but also for others in creating digital health interventions [9].

Therefore, this study reports findings from a personalized digital action planning process, focused on SSB and weight, that is embedded within the iSIPsmarter digital behavioral health intervention. The study aims are to (1) assess the frequency of action plan engagement, (2) evaluate participants’ perceived difficulty implementing their action plan and examine their progress toward achieving SSB reduction and weight goals, and (3) examine the selection of SSB- and weight-related barriers and strategies. This process evaluation corresponds to a prespecified secondary aim of the larger RCT focused on engagement with intervention components [20].

A recently published secondary analysis from the larger RCT examined the demand and implementation costs associated with iSIPsmarter’s stepped care engagement approach to promoting participant engagement in the digital intervention [22]. In contrast, this manuscript focuses specifically on engagement with the intervention’s action planning component. Findings are used to offer considerations for future integration of digital action planning components into behavioral interventions.

## Methods

### Study Design

#### Overview

This paper is an exploratory, longitudinal process evaluation of digital action planning embedded within the iSIPsmarter intervention. A 2-group RCT examined the efficacy of 2 internet-based programs aimed at decreasing SSB consumption [20]. Participants were randomized to receive one of two conditions: (1) iSIPsmarter (n=127), in which participants received 6 modules (ie, “Cores”) with personalized action planning and behavioral tracking of SSB and weight via SMS and cellular-enabled scales, or (2) a patient education website (n=122), in which participants had access to a static education site with no action planning and optional tracking of SSB and weight via paper diaries. Participants were enrolled in this study from July 2021 to December 2022. This paper focuses on process data from iSIPsmarter participants. While most digital action planning occurred in the first 9 weeks, extending the analysis through the 6-month assessment allowed for exploration of longer-term weight-related goal achievement.

#### Participants

The larger 2-group RCT recruited participants from Southwest Virginia and surrounding Appalachian counties using community-engaged, multimodal recruitment strategies. Recruitment efforts were conducted in partnership with a wide range of community stakeholders, including federally qualified health centers, local health districts, hospitals, local government agencies, and higher education institutions [23]. Recruitment strategies included both passive methods (eg, print materials, emails to listservs, web page postings, and press releases) and active methods (eg, community presentations and outreach events). Interested individuals completed an online eligibility screener, and eligible participants were subsequently contacted by study staff to complete informed consent and baseline assessment procedures. To be eligible for the study, participants were required to (1) be English-speaking, (2) be aged at least 18 years or older, (3) reside in Southwest Virginia or surrounding Appalachian counties, (4) consume more than 200 calories of SSB per day as assessed by a validated beverage intake questionnaire [24], (5) not participate in another program focused on energy balance–related behaviors, and (6) be willing to access an internet-enabled computer or tablet at least 1 time per week and receive SMS text messaging–based reminders. Participants included in this analysis were those who were randomized to receive the iSIPsmarter intervention and who had completed at least 1 iSIPsmarter Action Plan (ie, 2 Cores) (119/127, 94%).

#### iSIPsmarter Intervention Overview

In brief, iSIPsmarter was adapted from the original evidence-based SIP*smart*ER SSB intervention, which was informed by several phases of formative research and included three 90-minute in-person small-group classes and interactive voice response calls for behavioral self-monitoring and action planning [25,26]. Furthermore, guided by the Model for Internet Interventions [27] and best practices in human-centered design [28], a robust human-centered design process was used to adapt iSIPsmarter’s digital intervention structure, including the Cores, behavioral self-monitoring, and action planning [29]. The comprehensive 3-phase human-centered design process (contextual inquiry, prototype testing, and end user testing) spanned approximately 18 months. It involved a series of 13 semistructured, one-on-one interviews with 7 advisory team members from the targeted user group. Complete details of the intervention’s development and content are described elsewhere [20,29].

iSIPsmarter is a self-guided digital health intervention delivered via a secure web-based platform. Participants log in to iSIPsmarter with their email address and password to access 6 behavioral content Cores. Each Core is metered out to the user, with a new Core unlocked 7 days after completion of the previous Core. Therefore, while it is possible for Cores to unlock on a weekly schedule, the Cores do not always correspond to calendar weeks as participants may take longer than 1 week to complete a Core. Core content includes various media formats of text, audio, video, interactions, animations, and vignettes focused on SSB-related topics and skill building. Core content highlights the connection between SSB and weight outcomes through personalized interactions and messaging, which helps participants recognize how SSB reduction supports weight management and overall health. The Cores also provide tailored calorie and added sugar recommendations aligned with each participant’s weight-related goal. A complete list of each Core’s learning objectives is described elsewhere [20]. The program integrates digital behavioral tracking through SMS prompts and a cellular-enabled scale to support daily self-monitoring of SSB intake and weight. A personalized dashboard allows participants to view and track their progress, while the digital action planning process—embedded within 5 of the 6 Cores (ie, Cores 2 through 6)—guides participants through goal setting and problem-solving or coping planning (ie, barrier identification and identification of strategies to overcome barriers) with personalized feedback.

#### Behavioral Self-Monitoring

Throughout the intervention, participants engage in daily behavioral self-monitoring of both SSB and weight through an integrated system. iSIPsmarter delivers daily prompts for participants to report their total SSB intake in ounces for the previous day, offering the flexibility to receive these reminders via SMS text messaging, email, or both. Two-way SMS text messaging queries allow participants to reply directly with their SSB intake, which is automatically uploaded to their online iSIPsmarter dashboard. Alternatively, participants can manually enter their intake by signing into the program online. In addition, the daily email prompt encourages participants to weigh themselves using their cellular-enabled electronic BodyTrace scale and monitor their progress toward their weight goals. The BodyTrace scale automatically transmits weight data to the dashboard when a cellular connection is available, with the option for participants to manually log their weight online, if necessary.

#### Program Goals

Participants set a program goal in ounces for SSB in Core 2. Additionally, at the start of the intervention, participants select a weight-related reason for joining iSIPsmarter to either “lose weight” or “maintain weight.”

#### Action Planning With Personalized Feedback

**Figure 1.**
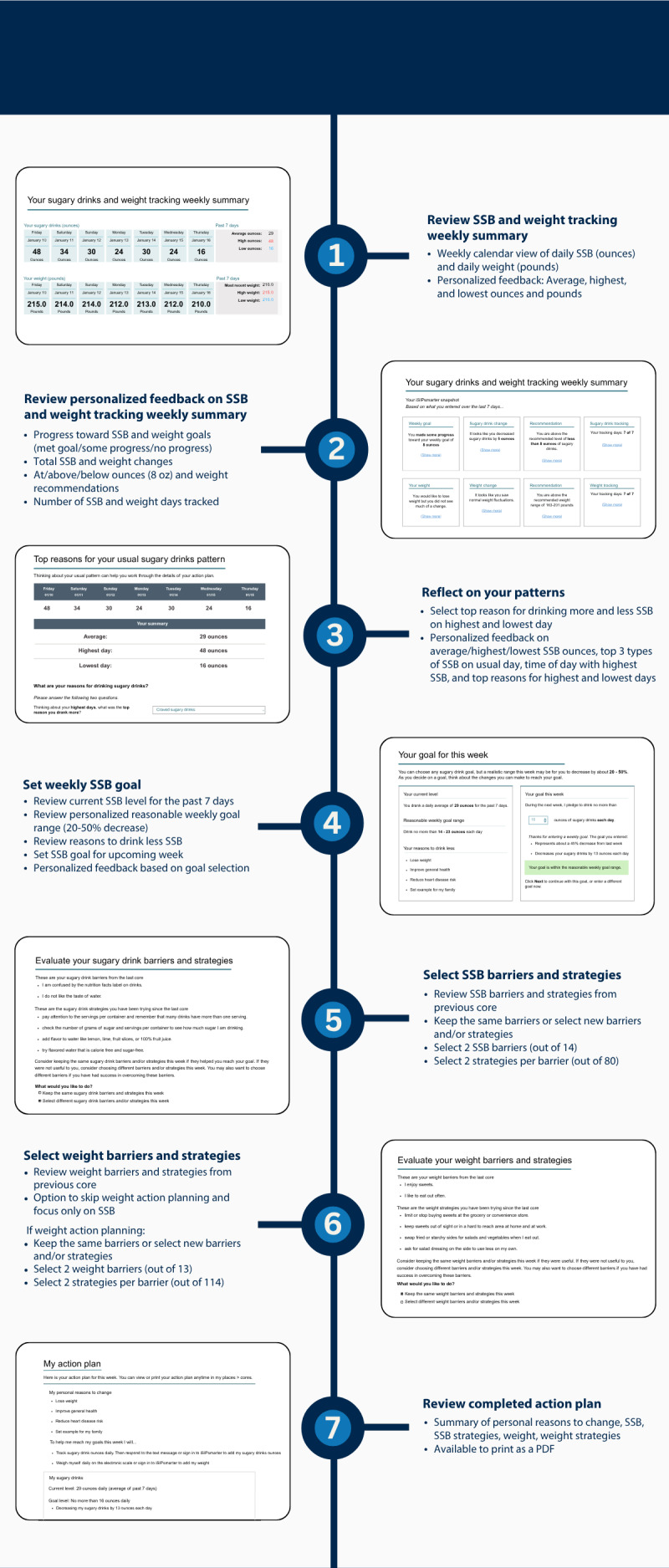
iSIPsmarter digital action planning process. SSB: sugar-sweetened beverage.

Throughout the program, participants can complete up to 5 personalized action plans. Action plans are embedded at the end of Cores 2 through 6. Thus, participants must complete an action plan in order to complete the Core. The iSIPsmarter action planning process was designed to provide automated, tailored behavioral support to participants to self-monitor and reduce their SSB intake and manage weight behaviors. During this structured process, participants set personal goals and identify barriers to making behavioral changes and strategies to overcome the barriers. Figure 1 provides a step-by-step overview of the action planning process.

As shown in Figure 1, the first step in the action planning process includes feedback on participants’ SSB behaviors and weight through a summary of tracking completed during the previous 7 days (step 1). Participants then receive personalized feedback on SSB and weight data (step 2), including progress toward SSB and weight goals, total SSB and weight change, comparisons to SSB and weight recommendations, and total number of days tracked. Participants are required to have at least 3 days of data to receive personalized feedback. Those with less than 3 days of data are prompted to manually enter data for up to the previous 7 days. Next, participants are encouraged to reflect on factors influencing SSB behaviors (ie, select a top reason for their highest and lowest SSB days), before receiving personalized feedback on their SSB patterns (step 3). Then, participants are encouraged to set a weekly goal to decrease their SSB in ounces by 20%‐50% from their previous week’s average (step 4); however, participants can choose a goal outside of the recommended range. This reduction target is repeated in subsequent action plans until the participant meets the recommendation of less than 8 ounces of SSB per day.

Participants’ achievement of their weekly SSB goal is assessed using their average intake during the 7 days preceding each Core. A participant’s goal is met if the average SSB ounces are less than or equal to the goal amount set in the prior Core. Some progress is achieved if the average SSB ounces are greater than the goal amount set in the prior Core but less than the average SSB ounces from the prior Core. No progress is achieved if the average SSB ounces are greater than the goal amount and greater than or equal to the average SSB ounces from the prior Core. If participants have less than 3 days of SSB diary data, they receive feedback that there are not enough data to determine progress toward their goal.

After setting their goals, participants are guided through a structured problem-solving process (step 5). They begin by selecting 2 SSB-specific barriers from a prepopulated list, followed by identifying 2 strategies per barrier to help overcome these challenges. To promote behavior change intentions, participants are prompted to complete the statement, “To help me reach my goal this week, I will...” and choose from a list of specific strategies mapped to each barrier.

The prepopulated barriers and strategies were intentionally designed to be specific and actionable, with many strategies incorporating key BCTs such as context cues and habit formation. The barrier and strategy lists were developed and tested through formative research to inform the original SIPsmartER intervention [26] and iterative end user testing to ensure that they were both culturally relevant and feasible for the intended population [29]. Barriers and strategies are prepopulated in drop-down menus to streamline decision-making and reduce participant burden, a critical consideration in internet-delivered interventions.

Participants also have the option to enter their own strategy if none of the listed options align with their needs, which prompts a free-text response field for personalized entry. In subsequent action plans, participants are shown their previously selected barriers and strategies and can choose to either maintain or revise them to reflect evolving challenges or preferences.

After participants select SSB-related barriers and strategies, they are offered the choice to select weight-related barriers and strategies or skip them if they want to focus exclusively on SSB. The weight action plan mirrors the SSB action planning process, in that participants identify 2 weight-related barriers and 2 corresponding strategies per barrier (step 6). Either skipping or selecting weight barriers and strategies concludes the action planning process for the Core. Thus, participants’ final action plan includes up to 4 self-selected barriers and up to 8 strategies to work on for the upcoming week. Once complete, the action plan is locked and cannot be changed (step 7). Participants can access their completed action plans from their website dashboard, and they can be printed as a PDF document.

In subsequent Cores, participants are prompted to rate how easy or hard it was for them to stick to the action plan they set in the previous Core. Tailored feedback is provided based on their response. For example, participants who report that their action plan was impossible or hard to follow are encouraged to choose smaller weekly goals and to select different strategies to support their success.

### Measures

#### Overview

Demographic data were self-reported at baseline via an online questionnaire. Participants’ zip codes and the rural-urban continuum codes (RUCC) were used to determine rurality status. The codes range from 1 to 9, with higher values indicating more rural areas [30]. Process data for each participant, including action planning goals and goal achievement, perceived action planning difficulty, barrier and strategy selections, SMS text messaging–based and manual SSB tracking entries, and weight data transmitted through cellular-enabled scales or manually entered online, were automatically captured through the iSIPsmarter web-based administrative platform. Baseline and 6-month follow-up data were exported from the administrative platform for analysis.

#### SSB Action Planning

Participants’ action planning data included self-selected personal reasons to change, summaries of SSB behavioral self-monitoring, a program SSB goal, and weekly SSB goals (all SSB summaries and goals were in ounces).

#### Participants’ Perceived Difficulty Implementing Their Action Plan

Participants’ perceived difficulty implementing their action plan was assessed at the start of Cores 3‐6. Perceived difficulty was not assessed in Core 2 because participants did not complete an action plan in Core 1. The question, “How hard or easy was it to stick to your action plan this past week?” was rated on a 5-point Likert scale ranging from (0) “impossible” to (4) “very easy.”

#### SSB Goal Achievement

Participants received feedback on their weekly goals, which included (1) goal achieved, (2) some progress toward the goal, (3) no progress toward the goal, and (4) not enough diary data to provide feedback.

#### SSB-Related Barriers

Data included participants’ self-selected SSB barriers for each completed action plan. For each action plan, participants selected 2 barriers from a list of 14 SSB-related barriers. See the Results section for the complete list.

#### SSB-Related Strategies

Within each SSB barrier, participants selected 2 strategies. Strategies were mapped to barriers and ranged from a total of 4‐9 per barrier, with a total of 80 possible strategies.

#### Weight Action Planning

Participant weight was measured via a cellular-enabled BodyTrace scale. Participants’ weight action planning data included a program weight goal (ie, lose or maintain weight), which was set in Core 1.

#### Weight Goal Achievement

Participants’ percent weight change was assessed from baseline to 9-week and baseline to 6-month follow-up assessments. The program weight loss goal was achieved if the participants lost at least 1 pound. The program weight maintenance goal was achieved if the participants’ weight change was less than 1 pound [31].

#### Weight-Related Barriers

Participants selected 2 weight-related barriers from a list of 13. See the Results section for the complete list.

#### Weight-Related Strategies

Participants selected 2 weight-related strategies per barrier. Possible strategies to choose from for each barrier ranged from 8 to 13, with a total of 115 possible strategies.

### Data Analysis

Data analysis was conducted using SPSS (version 29.0; IBM Corp). Descriptive statistics were used to calculate frequencies and percentages at each Core for action plan completion, perceived difficulty implementing action plans, and SSB and weight goal achievement. SSB and weight barrier and strategy frequencies were aggregated across all completed action plans.

### Ethical Considerations

This study was approved by the University of Virginia Institutional Review Board (IRB-HSR 22130) and was also registered with the ClinicalTrials.gov registry: NCT05030753. All participants provided written informed consent prior to enrollment. Participants were allowed to withdraw from the study at any time during the research process. All data used in this analysis were deidentified to ensure participant privacy and confidentiality. Participants received compensation in the form of a cellular-enabled scale at baseline and gift cards (US $50) at the follow-up assessments.

## Results

### Sample and Demographics

Of the 127 enrolled iSIPsmarter participants, 119 (94%) participants completed at least 1 action plan and are included in this analysis. As shown in Table 1, most participants were Caucasian, female, and from more rural counties (ie, RUCC 3=54, 45%; RUCC 4‐9=52, 44%), with an average age of 42 (SD 11.9) years. Just over half were college educated and nearly half had household incomes less than US $55,000 per year. SSB intake at baseline averaged 43.2 (SD 23.6) fluid ounces. Notably, 27 (23%) of participants were overweight and 74 (62%) had obesity.

**Table 1.** Baseline characteristics of iSIPsmarter intervention participants (N=119).

Baseline characteristics	Participants
Race, n (%)	
White (Caucasian)	106 (89.1)
African American	8 (6.7)
>1 race	2 (1.7)
Other	3 (3)
Ethnicity, n (%)	
Hispanic/Latino and Latina	3 (2)
Sex, n (%)	
Male	23 (19.3)
Female	96 (80.7)
Age (years), mean (SD)	42 (11.9)
Age (years), n (%)	
18‐24	7 (5.9)
25‐44	56 (47.1)
45‐64	53 (44.5)
≥65	2 (1.7)
Education, n (%)	
High school graduate or less	13 (10.9)
Some college	40 (33.6)
College graduate	27 (22.7)
Graduate school	39 (32.8)
Annual household income (US $), n (%)	
14,999 or less	8 (6.8)
15,000‐$34,999	24 (20.5)
35,000‐$54,999	25 (21.4)
≥55,000	60 (51.2)
Rural-Urban Continuum Code, n (%)	
1‐2 (medium-large metro)	13 (10.9)
3 (small metro)	54 (45.4)
4‐9 (nonmetro)	52 (43.7)
SSB^a^, mean (SD)	
SSB intake, fluid ounces	43.2 (23.6)
BMI, n (%)	
Underweight	1 (0.8)
Normal	17 (14.3)
Overweight	27 (22.7)
Obesity	74 (62.2)

a SSB: sugar-sweetened beverage.

### SSB Action Plan Engagement

Participants completed on average 4.5 (SD 1.1) out of 5 SSB action plans. Furthermore, of the 119 participants who completed at least 1 action plan (Core 2), 95 (80%) completed all 5 (Cores 2‐6). Action plan completion declined slightly over time, with 113 (95%) participants completing Core 3, 106 (89%) completing Core 4, and 101 (85%) completing Core 5. This pattern mirrors the overall decline in Core completion across the intervention.

### Participants’ Perceived Difficulty Implementing Their Action Plan

**Figure 2.**
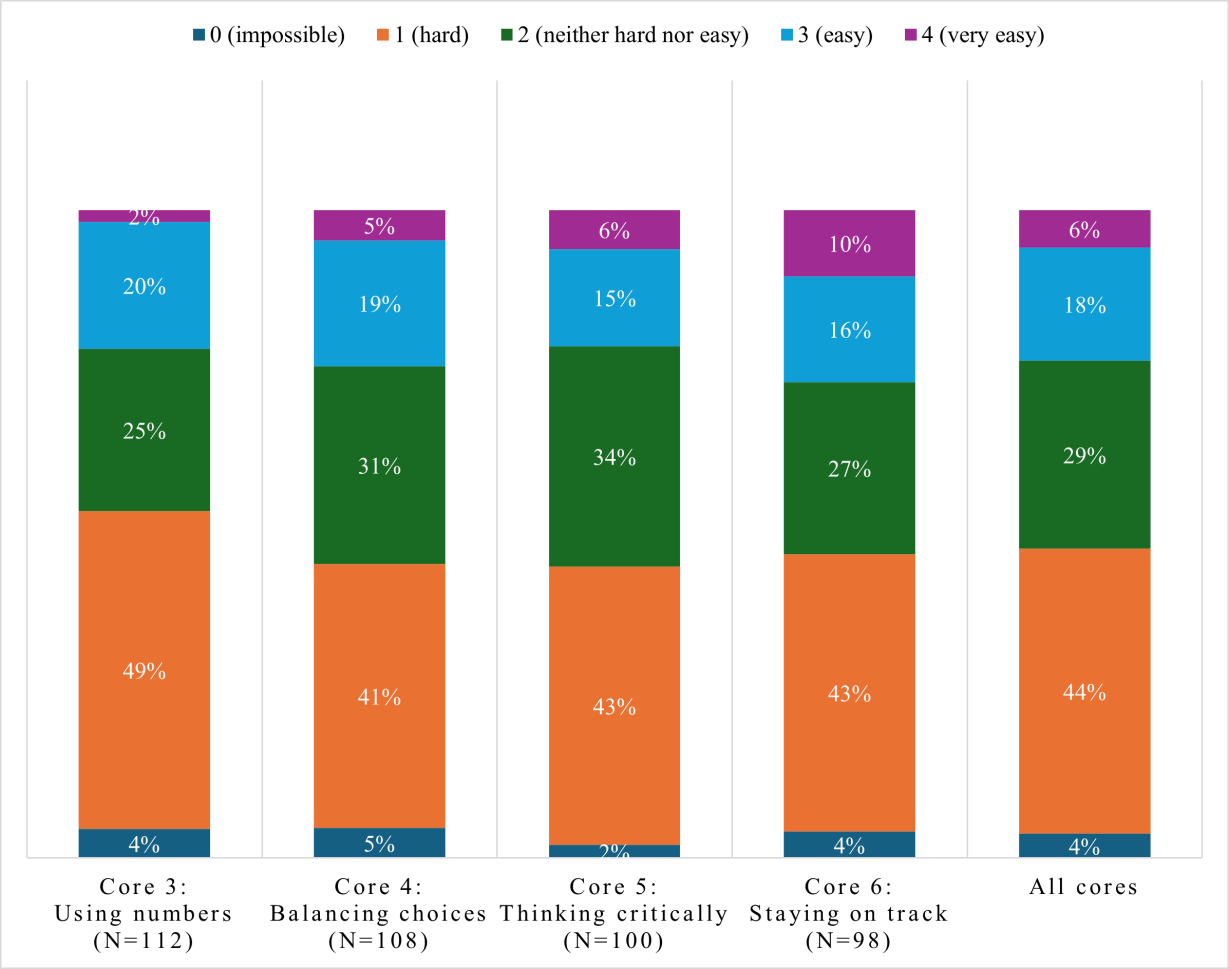
Participants’ perceived difficulty implementing their action plan.

As shown in Figure 2, across all Cores, participants’ perceived difficulty implementing their action plan remained relatively stable. On average, 48% rated the tasks as impossible or hard, 29% as neither hard nor easy, and 24% as easy or very easy.

### SSB Goal Achievement

**Figure 3.**
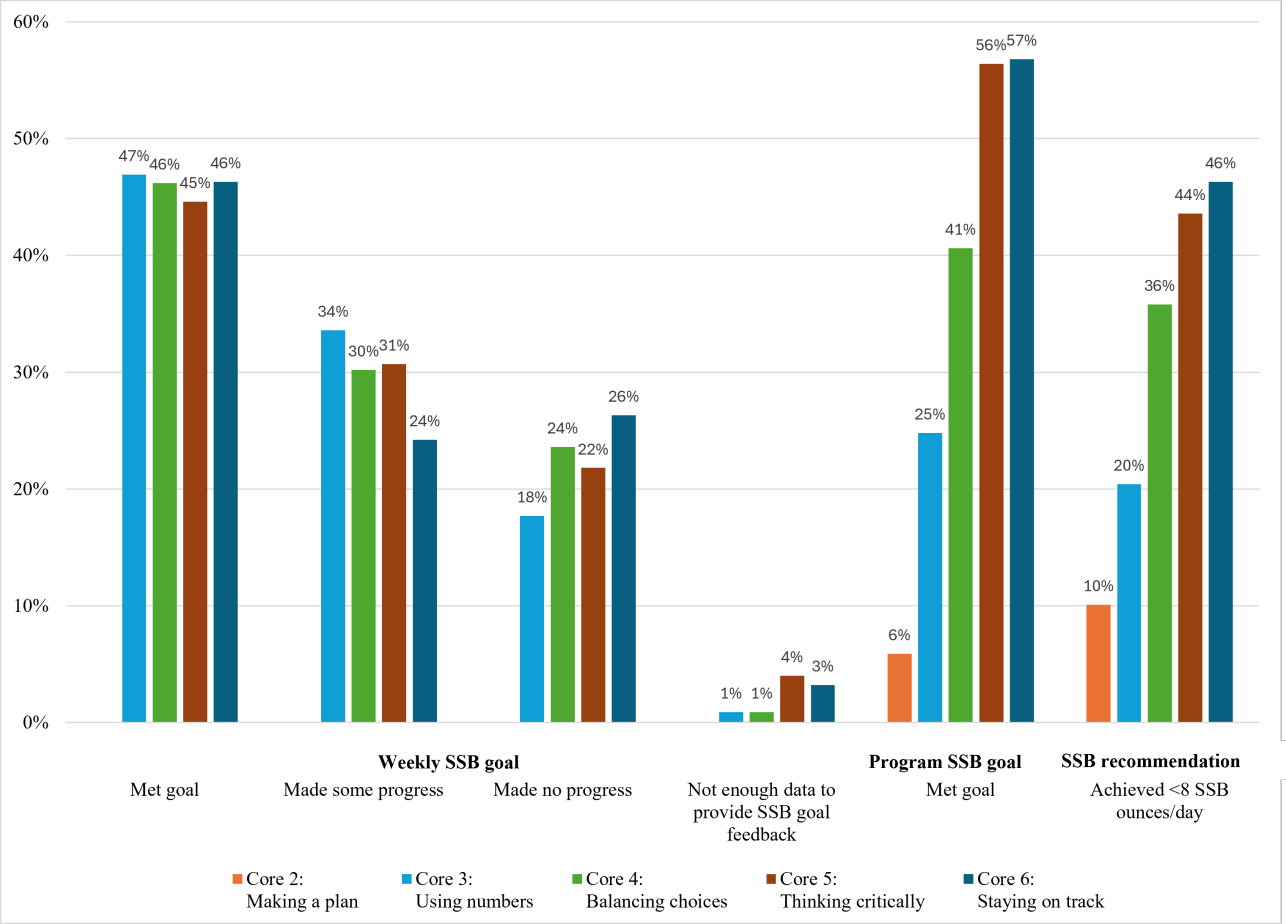
Participants’ progress in achieving SSB goals and recommendations. SSB: sugar-sweetened beverage.

Across each of the Cores, Figure 3 illustrates participants’ progress toward three SSB goals: (1) their weekly goal, (2) their program goal, and (3) achievement of <8 ounces of SSB per day recommendation. Regarding weekly SSB goals, the percentage of participants meeting their goals remained relatively stable across the Cores, averaging about 45%. Also, about one-third of participants made some progress toward their weekly goals at each Core. Meanwhile, there was a steady increase in the proportion of participants who reduced their daily SSB intake to achieve the recommendation of <8 ounces per day. At the end of Core 6, 57% (54/95) of participants met their program goal and 46% (44/95) achieved the recommendation of <8 ounces of SSB per day.

### SSB Barrier and Strategy Selection

As shown in Table 2, 45% (53/119) of participants selected different SSB barriers at least once during the action planning process, and 53% (63/119) of participants revised their chosen strategies at least once. On average, participants selected 2.8 (SD 1.1) unique barriers and 6.4 (SD 2.9) unique strategies. As shown in Table 3, the most common reported barriers to reducing SSB were (1) needing caffeine from SSB, (2) not wanting to give up the taste of SSB, (3) SSB being a habit, (4) not liking the taste of no-calorie sugar substitutes, and (5) needing energy from SSB. The top five reported strategies to reducing SSB were as follows: (1) reduce my caffeinated SSB slowly to avoid caffeine withdrawal, (2) replace sugary caffeinated drinks with nonsugary caffeinated drinks, (3) get more sleep to feel less tired and dependent on caffeinated SSB, (4) slowly reduce SSB to tame my sweet tooth, and (5) use the iSIPsmarter Diary to track SSB and learn my habit.

**Table 2.** Summary of SSB^a^-related and weight-related barriers and strategies.

	SSB-related barriers and strategies (n=119)	Weight-related barriers and strategies (n=112)
Participants who changed barriers, n (%)	53 (45)	69 (62)
Number of unique barriers selected, mean (SD) (possible range 2‐10)	2.8 (1.1)	3.0 (1.0)
Participants who changed strategies, n (%)	63 (53)	82 (73)
Number of strategies selected, mean (SD) (possible range 4‐20)	6.4 (2.9)	6.6 (2.7)

a SSB: sugar-sweetened beverage.

**Table 3.** Frequency of iSIPsmarter SSB^a^-related barriers selected across all completed iSIPsmarter action plans and participants.

Barrier	Frequency
Need the caffeine from SSB	212
Do not want to give up the taste of SSB	193
SSBs are a habit	139
Do not like the taste of no-calorie sugar substitutes	98
Need the energy from SSB	97
Drink SSB with family or friends	88
Do not like the taste of water	88
Have too many SSBs around	45
Concerns about no-calorie sugar substitutes	44
Hard to choose smaller sizes	43
Buy SSB because they are low-cost	14
Concerns about water safety	4
Persuaded by advertising	3
Confused by the nutrition facts label	1
Total	1069

a SSB: sugar-sweetened beverage.

### Weight Action Plan Engagement

Participants completed on average 3.3 (SD 1.5) weight action plans, with 94% (112/119) of participants completing at least 1 weight action plan. Engagement in weight action planning varied, as 6% (7/119) of participants skipped weight action planning, 7% (8/119) completed 1 weight action plan, 24% (29/119) completed 2 weight action plans, 7% (8/119) completed 3 weight action plans, 29% (34/119) completed 4 weight action plans, and 28% (33/119) completed all 5 weight action plans.

### Weight Goal Achievement

**Figure 4.**
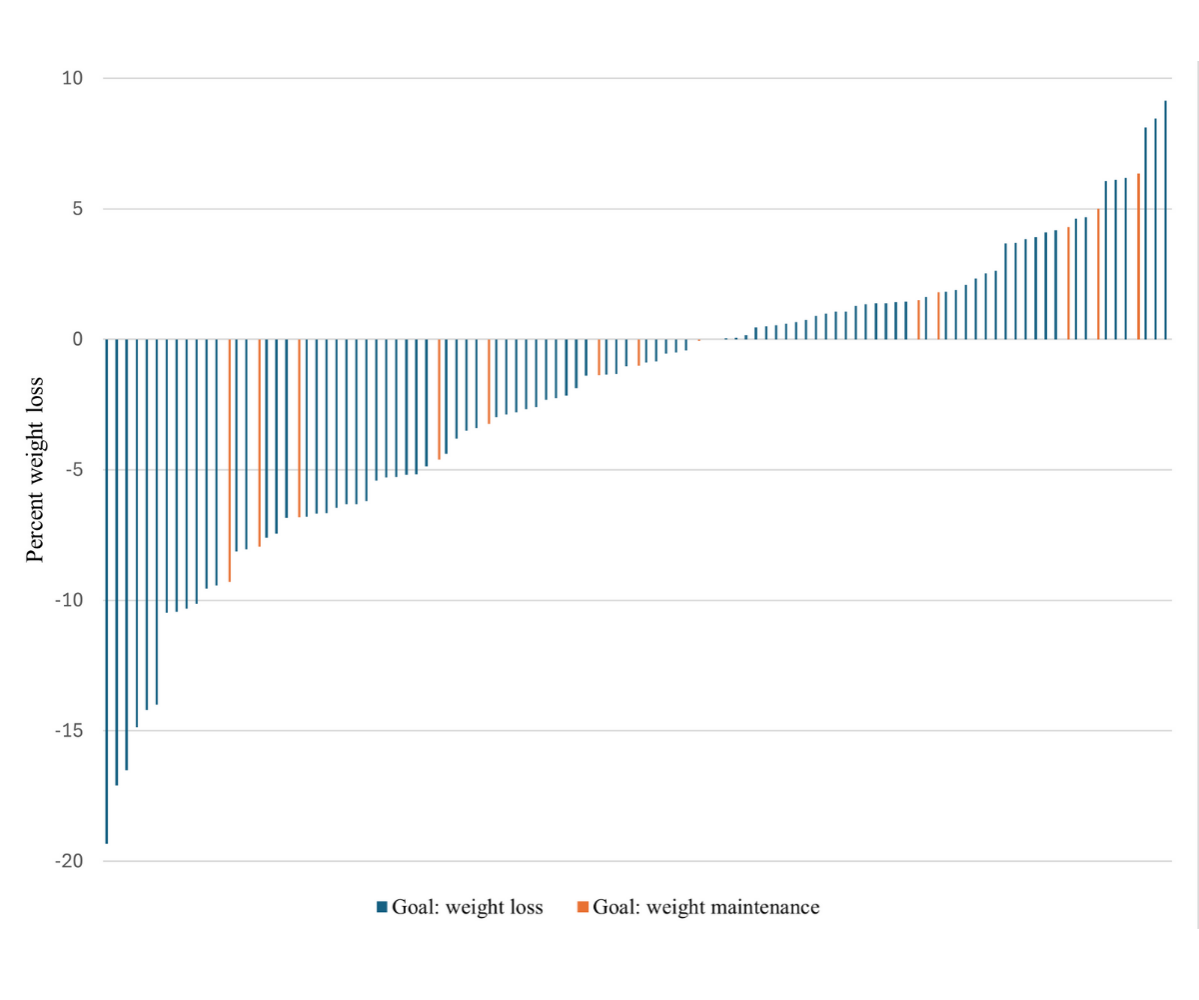
Participants’ progress in achieving program weight goal at 6 months.

At the beginning of the intervention, 86.6% (103/119) of participants set a weight goal to lose weight. Among these participants with follow-up weight data, the average weight loss was −1.3% (SD 2.6) at the 9-week follow-up, with 61% (57/94) achieving their goal. By 6 months, weight loss increased to −2.1% (SD 5.6), with 54% (49/90) achieving their goal (Figure 4).

The other participants (16/119, 13.4%) set a goal to maintain weight during the intervention. Among these participants with follow-up weight data, 7% (1/14) achieved their goal at the 9-week follow-up, and 8% (1/13) achieved their goal at the 6-month follow-up. At 6 months, the average weight change was −1.2% (SD 5), with most participants losing, rather than maintaining weight (Figure 4).

### Weight Barrier and Strategy Selection

As illustrated in Table 2, 62% (69/112) of participants revised their weight barriers throughout their action planning process, and 73% (82/112) of participants updated their weight-related strategies. As shown in Table 4, the most common reported weight-related barriers were (1) enjoying sweets, (2) struggling with portion sizes, (3) being too busy or tired to eat healthy foods, (4) having difficulty staying motivated, and (5) snacking throughout the day. The top five reported weight-related strategies were as follows: (1) limit or stop buying sweets at the grocery or convenience store; (2) preportion my serving when I have sweets, and not eat straight from the package; (3) keep sweets out of sight or in a hard to reach area at home and at work; (4) replace sweet treats with fruit, such as berries, melon, or apples; and (5) ask myself if I really need a second helping and wait 20 minutes before deciding.

**Table 4.** Frequency of iSIPsmarter weight-related barriers selected across all completed iSIPsmarter action plans and participants.

Barrier	Frequency
Enjoy sweets	198
Struggle with portion sizes	101
Too busy or tired to prepare healthy foods	87
Eat out often	86
Hard time staying motivated	61
Snack throughout day	59
Enjoy high-fat dairy	51
Enjoy high-fat meats	43
Enjoy salty snacks	36
Healthy foods cost a lot of money	27
Hard time eating fruits and vegetables	23
Drink calories through alcohol	9
Do not like the taste of healthy foods	1
Total	782

## Discussion

### Overview

This is one of the first studies to thoroughly explore a digital action planning process and to conduct a comprehensive process evaluation of a nutrition-focused digital behavioral health intervention [3,9,26,32]. In contrast to existing digital interventions that mention action planning superficially or as a single feature, the iSIPsmarter intervention incorporates it as a multistep, iterative process aligned with behavioral theory and delivered through personalized, user-guided digital Cores. This level of integration and process evaluation contributes to the much-needed understanding of how key BCTs are operationalized in digital formats. Findings from our study contribute to the growing body of literature on digital behavioral health interventions, emphasizing the value of structured and personalized goal-setting and action planning strategies in supporting self-regulatory behaviors, such as SSB intake and weight management.

### Principal Results

#### SSB Action Planning

Findings from this study highlight the usefulness of embedding a low-cost, scalable action planning process within a digital behavioral health intervention. The iSIPsmarter action planning component provided automated, tailored support to help participants monitor their SSB intake, set goals, identify barriers, and select personalized strategies. Engagement was high, with 80% (95/119) of participants completing all 5 action plans. iSIPsmarter’s action planning process allowed the user to create new action plans or repeat previously made plans to reinforce them. High engagement across all 5 action plans aligns with recent research indicating that the number of action plans is positively associated with improved health behaviors, with the strongest associations observed when individuals create 5 plans [7]. Furthermore, high engagement with iSIPsmarter’s action planning process underscores the program’s usability and key design features, which were adapted and developed from our prior efficacious SSB intervention [26] and using best practices in human-centered design [28,29]. Findings from this study are especially meaningful, given its rural context, in which rural populations often encounter persistent barriers to accessing face-to-face behavioral health interventions. This suggests that well-designed, digitally delivered behavior change interventions can extend the reach of behavioral programs to underserved communities.

#### Participants’ Perceived Difficulty Implementing Their Action Plan

Reducing SSB intake is a complex and difficult behavior to change. It is often embedded in daily routines and driven by habit, preference, and societal norms. Within this study, participants’ perceived difficulty in implementing their weekly action plans reflected this challenge. Across much of the program (Cores 3‐6), participants rated implementing their action plan “hard” most often, with only a small number of participants rating their plans “impossible” or “very easy.” These difficulty ratings closely mirrored goal achievement patterns. Importantly, those who rated their plans as difficult continued to engage in the action planning process. This persistence speaks to the value participants placed on the intervention and its relevance to their goals. This suggests that well-designed digital behavior change interventions can sustain engagement and support progress, even when the target behavior is difficult. Future research should use data capturing participants’ perceived difficulty to provide real-time tailoring, with added support for those consistently reporting difficulty sticking to their action plan.

#### SSB Goal Achievement

Our study findings illustrate an encouraging trend in participants’ progress toward reducing SSB intake. While the proportion of participants meeting their personalized weekly SSB goal was stable at about 46% across the action planning process, an additional 33% made some progress toward their weekly goals. Together, 79% of participants made progress each week, which reflects a high level of progress, especially given the complexities of changing SSB behaviors. Goal attainment rates align with other behavioral nutrition interventions, where about half of the participants meet their goals [33].

Furthermore, there was a notable, steady increase in those reducing their SSB intake to the recommended less than 8 ounces. By the final action plan, nearly half of the participants had met this recommendation. Although nationally representative data typically report SSB intake by frequency or total added sugars intake, available estimates suggest that on a given day, US adults consume approximately 145 calories from SSB [34], which is approximately one 12-ounce serving of soda. Thus, very few US adults meet the less than 8-ounce recommendation. The achievement of this recommendation among iSIPsmarter participants is especially encouraging, given that participants’ average SSB intake at the start of the intervention was approximately 43 ounces per day, equivalent to more than 3½ cans of soda.

#### SSB-Related Barriers and Strategies

Over half of the participants maintained the same 2 SSB-related barriers throughout the action planning process, indicating consistency in perceived challenges. The most frequently cited barriers (ie, the need for caffeine, reluctance to give up the taste of SSBs, and the habitual nature of SSB consumption) are consistent with findings from prior SSB-related literature across diverse populations [35,36]. However, over half of the participants modified their strategies associated with these goals at least once during the action planning process. This adjustment in strategy selection may reflect participants’ efforts to identify the most effective approaches for overcoming barriers, which are likely to change over time. This finding highlights the importance of flexibility in goal setting and troubleshooting barriers through repeated action planning. Moreover, research has shown that counterhabitual actions are important for intentions to be effective in breaking strong habits [7]. In the case of iSIPsmarter, strategies were tailored to barriers and were structured so that they could facilitate the deconstruction of ingrained SSB consumption barriers.

The most frequently selected SSB strategies–such as gradually reducing caffeine intake, substituting nonsugary caffeinated drinks, improving sleep to reduce SSB caffeine dependency, and using tracking tools such as the iSIPsmarter Diary—highlight the importance of providing users with multiple strategies to reduce SSB consumption. These strategies target both the physical drivers of SSB consumption, such as caffeine dependence and taste preferences, and behavioral factors, such as habit formation and building self-awareness through behavioral self-monitoring. These strategies have been shown to be key BCTs in behavioral health interventions focused on improving dietary behaviors [37].

While some research has emphasized the potential benefits of self-constructed plans, this approach can introduce participant burden, especially in digital settings without human-delivered support. To balance personalization with usability, iSIPsmarter offered an evidence-informed list of barriers and strategies developed through prior evidence-based research and rigorously tested using a user-centered design approach [26,29]. Furthermore, a free-text “use my own strategy” option was available but was used infrequently, suggesting that most participants preferred and were able to identify their strategies using the structured list. This design may reduce cognitive load while still allowing for personalization, particularly among populations with limited health literacy or digital experience. Developing and testing intervention components, such as barriers and strategies, from the end user’s perspective is recognized as a critical element in the design and implementation of digital behavioral interventions [38].

#### Weight Action Planning

The varying levels of participant engagement with the weight action planning component provide insights into how optional aspects of an intervention may influence participation. While the majority (112/119, 94%) of the participants completed at least 1 weight action plan, fewer (33/119, 28%) completed all 5 weight action plans. Although 87% (103/119) of the participants identified weight loss as an initial goal, engagement with weight action planning diminished over time. Reasons for this decline in weight action planning are not entirely clear, yet it is plausible that some participants may have experienced weight-related information avoidance or negative emotions with weight self-monitoring and action planning [39,40]. Furthermore, the weight action planning patterns may suggest that participants were selective, possibly prioritizing the SSB-focused components of the intervention over the weight-specific ones. The ability to choose between SSB-related and weight-related barriers likely allowed for a more customized approach but may have also led participants to limit engagement with optional elements when those did not align as closely with their perceived barriers. This selective engagement reflects the need for flexibility in interventions, as well as for further examination of how weight self-monitoring can best be structured to support engagement over time. Given that 85% (101/119) of the enrolled iSIPsmarter participants were with overweight or obesity, compared with the estimated national prevalence of 74%, the need for future research efforts to promote engagement in weight action planning is especially critical in Appalachia and other regions facing similar weight disparities [41].

#### Weight Goal Achievement

Participants’ weight-related goal achievement underscores iSIPsmarter’s success in supporting weight loss goals. Over half of the participants who set a weight loss goal successfully met it, demonstrating the intervention’s potential to support meaningful behavior change. By 6 months, the average percent weight loss was nearly double among those with a weight loss goal compared with those with a weight maintenance goal. Additional opportunities for future research are to explore the relationship between engagement with weight action planning components and the achievement of weight-related goals.

#### Weight-Related Barriers and Strategies

The high proportion of participants who changed their weight-related barriers and strategies illustrates a dynamic process of problem-solving and coping planning. In contrast to SSB-related barriers, participants appeared to face more variable challenges related to weight. The most common weight-related barriers—enjoying sweets, struggling with portion sizes, and being too busy or tired to eat healthy foods—reflect common and complex challenges in weight management [42,43]. The top barrier of sweets reveals participants’ affinity for all sugar and should be considered for future SSB interventions incorporating nutrition-related content. Frequent updates to weight-related strategies suggest that participants engaged in action planning as an ongoing, iterative process rather than a one-time exercise. Notably, most participants selected new strategies at least once, indicating that repeated action planning may promote more flexible and personalized approaches to overcoming barriers. This pattern of strategy modification may reflect participants’ efforts to fine-tune their weight-related behaviors through trial and error, adapting their approaches in response to evolving challenges and contexts, as well as instances where previous strategies were unsuccessful. The most reported strategies, such as limiting or stopping the purchase of sweets, preportioning servings, and replacing sweet treats with fruit, are practical and actionable steps that participants can take to modify their eating habits. The emphasis on portion control and environmental modifications (eg, keeping sweets out of sight) aligns with behavioral strategies to support sustainable weight management [44].

### Comparisons With Prior Work

While prior research has established the importance of action planning as a BCT, detailed reporting on how action planning is operationalized and reported across digital interventions remains limited. Many studies reference action planning without describing how plans are structured, how frequently they are completed, or the extent to which plans are personalized and adapted over time [3,32,45]. This limits the ability to compare digital health interventions and understand which aspects of digital action planning best support behavior change.

A recent umbrella review underscores this gap in reporting. Although a small subset (ie, 41/388, 11%) of digital health intervention studies used action planning, descriptions of action plan completion varied widely, and details regarding the number of action plans created were often overlooked [9]. Among those who did report frequency, more than half relied on a single action plan throughout the intervention period, despite evidence that goal setting and action planning are most effective when implemented at multiple time points and reevaluated with personalized feedback [46].

Our study helps address these gaps by providing detailed process data on a multitimepoint, digitally delivered action planning process within a nutrition-focused intervention. Without this level of detail in the literature, it is difficult to replicate successful digital action planning strategies across health domains and populations. Moreover, although evidence supports the effectiveness of digital action planning across multiple health domains, there is a dearth of literature on digital action planning in nutrition-related behavioral interventions [9].

### Limitations

Several limitations should be considered when interpreting study findings. First, generalizability may be limited by the study population and targeted rural Appalachian region. Second, reliance on self-reported SSB intake data may introduce recall bias, potentially affecting the accuracy of reported consumption levels. Third, due to the exploratory nature of this paper and the absence of hypothesis testing, only descriptive statistics were used, which may limit interpretations. Although there may be interesting questions regarding associations among the reported descriptive variables, this study was not designed or powered to test inferential relationships, and attempting to do so could introduce analytical error and interpretations. Fourth, while participants rated their perceived difficulty sticking to their action plans, they did not explicitly rate whether they executed their action plan. This is an important area of future research. Finally, while the study reports on engagement with action planning, it does not explore user perceptions or qualitative feedback on the digital action planning experience. Perceptions of participants’ experience with the iSIPsmarter intervention, including barriers to adherence, usefulness, and perceived impact, are explored elsewhere [47]. These limitations should be considered in the context of the study’s strengths, which include (1) leveraging a digital health intervention to gather rich, longitudinal data on participants’ engagement with digital action planning, (2) using digital technologies to collect SSB and weight data for self-monitoring and personalized feedback, and (3) the inclusion of a comprehensive set of prepopulated barriers and strategies informed by prior research.

### Future Research

Our study highlights several important areas for future research. First, future studies should capture adherence to selected action plan strategies, perhaps through ecological momentary assessment methods [48]. Ecological momentary assessment could provide valuable real-time insights into when and why action plans are or are not enacted and provide opportunities to support implementation intentions. Second, future research should examine the optimal number of action plans, barriers, and strategies, as well as the impact of prepopulated versus open-ended (write-in) options on participant engagement and behavior change. These studies could help determine the best balance between minimizing participant burden and maximizing intervention effectiveness. Third, researchers should investigate the long-term effects of digital action planning on maintaining behavior changes beyond the intervention period. Fourth, future research should evaluate the scalability and adaptability of digital action planning strategies in diverse populations, including those with varying socioeconomic, cultural, and geographic contexts. Such efforts will be critical for optimizing digital action planning strategies and maximizing their potential to support sustainable health behavior change across diverse settings. Additional considerations for digital action planning in behavioral health interventions are provided in Textbox 1.

Textbox 1. **Considerations for digital action planning in behavioral health interventions.**

Although action planning has been identified as a key behavior change technique, there are a limited number of studies using digital action planning in digital health interventions. Moreover, few studies report on key design features or key process data from action planning. As such, we offer four considerations for developing digital action plans for digital health interventions:

1. *Leverage formative data and human-centered design processes to inform the development of digital action planning components*: Integrating formative data (eg, qualitative interviews or focus groups with intended users) with human-centered design processes can support the development of structured, preprogrammed action planning components that are relevant, realistic, and accessible to the intended users. In digital interventions, this approach helps balance personalization with usability and scalability, reduces participant burden, and supports sustained engagement by ensuring that action planning processes are culturally relevant and responsive to user needs.
2. *Capture and transparently report process-level engagement data*: Descriptive data on action plan utilization offer valuable context for interpreting participant engagement with an intervention. Furthermore, reporting participants’ perceived difficulty, goal progress, and strategy modification can help researchers understand how individuals interact with action planning components over time and support replication of successful digital action planning processes.
3. *Digital action planning strategies should be personalized and incorporate feedback on self-monitoring behaviors*: Personalized feedback on self-monitoring behaviors provides a tailored approach to supporting users in achieving and sustaining behavior change goals. This approach can enhance motivation and engagement and reduce barriers to making changes. Digital action plans that tailor feedback to the user provide personalized insights into strategy modifications needed to maintain or improve behaviors, ultimately leading to more successful and sustainable behavior change outcomes.
4. *Optimize data collection to align with the intervention’s budget and constraints*: Digital action planning offers unique opportunities to not only collect and analyze individual-level process data but also structure interventions in novel ways, such as adaptive interventions and those with personalized, tailored feedback. Ultimately, the budget, scope, and technical capabilities will inform how digital action planning can be structured and how much data can be collected. Researchers and interventionists should consider the cost-benefit of developing action planning systems that can be replicated across multiple health behaviors and behavioral interventions.

### Conclusions

This research provides a detailed process evaluation of digital action planning as a core BCT within a nutrition-focused digital behavioral health intervention. Specifically, the findings highlight the usefulness and strong participant engagement with a structured, digitally personalized goal setting and action planning process. Participants’ high utilization of action plans, frequent strategy updates, and steady progress toward SSB and weight goals suggest that action planning was useful. This is especially relevant for underserved populations where access to preventive health care is limited. By embedding action planning alongside other self-regulatory BCTs—such as goal setting, self-monitoring, and personalized feedback—iSIPsmarter created a cohesive digital behavior change framework that allowed participants to adapt their SSB and weight-related strategies over time. These findings reinforce the importance of designing digital action planning components that are user-centered and responsive to individual needs and contexts.

As digital behavioral health interventions continue to expand, this study underscores the value of elevating action planning from a passive or single-use BCT to a central, iterative component that drives engagement and behavior change. Researchers and interventionists interested in digital action planning can use findings from this study and considerations for digital action planning to integrate effective action planning components into their digital behavioral health interventions. Although focused on a nutrition behavior, it may provide insights into digital action planning strategies that can be applied across various health domains and behaviors. Overall, this study contributes to the growing body of literature on digital action planning as a key BCT and highlights its potential for promoting health behavior change in underserved populations.

## Abbreviations

BCT: behavior change technique
RCT: randomized controlled trial
RUCC: rural-urban continuum code
SSB: sugar-sweetened beverage
